# Enterococcus faecalis Infective Endocarditis Associated With Colorectal Cancer

**DOI:** 10.7759/cureus.55096

**Published:** 2024-02-27

**Authors:** Abdullah Alziyadi, Mohmmad Al Adwani, Hamma Abdulaziz, Rawan Aloufi, Amjad Althagafi, Abeer Alsulaimani, Abdulaziz Alotaibi

**Affiliations:** 1 General Surgery, Al Hada Armed Forces Hospital, Taif, SAU; 2 Colorectal Surgery, Al Hada Armed Forces Hospital, Taif, SAU

**Keywords:** colorectal cancer, colonoscopy, malignancy, enterococcus faecalis, infective endocarditis

## Abstract

Enterococcus faecalis (E. faecalis) is considered the third most common source of infective endocarditis. Some of the published reports linked its origin to colorectal cancer. We report a 70-year-old male patient diagnosed with E. faecalis infective endocarditis complicated by myocardial infarction. The patient also experienced symptoms of melena and anemia, prompting a colonoscopy. A colon mass was found and a biopsy revealed adenocarcinoma. The patient underwent a left hemicolectomy. In addition to that, he was treated for his cardiac issues. Many studies suggest screening for colonoscopy in patients with E. faecalis infective endocarditis to investigate its origin and potential association with colorectal cancer.

## Introduction

There is a strong association between Streptococcus bovis (S. bovis) infective endocarditis and colorectal cancer (CRC) [1]. They found that the prevalence of CRC in patients with S. bovis reaches up to 60% [2]. In these patients, routine colonoscopy is recommended by American [3] and European [4] guidelines. Staphylococcus aureus is now the most prevalent cause of infective endocarditis at about 26.6% of all cases, followed by viridans group streptococci at 18.7%, and Enterococcus faecalis (E. faecalis) the third most common source of infective endocarditis, responsible for 5% to 15% of the cases. The source of bacteremia in most cases is undetermined and if identified, is mostly related to the genitourinary system. Some of the published reports linked its origin to hidden colorectal cancer. Here we report a case of E. faecalis associated with CRC.

## Case presentation

A 70-year-old male patient presented to the emergency room (ER) in Al Hada Armed Forces Hospital, Taif, Saudi Arabia, complaining of shortness of breath, on and off, for a day in relation to movement, palpitations and melena. However, there was no history of chest pain or other gastrointestinal symptoms. On examination, the patient was conscious, fully oriented, and vitally stable. His abdomen was soft and lax with tenderness over the suprapubic region. Per rectal examination revealed melena and no palpable mass. 

His labs revealed microcytic anemia and a blood transfusion was started in the ER with two units of packed red blood cells (PRBCS). Also, there was a marked elevation of troponin, and an electrocardiogram (ECG) showed ST depression in leads V4-V6. The patient was admitted to the cardiology department for more investigation.

From the gastroenterology side, the patient underwent a colonoscopy to evaluate the source of bleeding. It revealed an infiltrating ugly, fungating, irregular, and friable mass about 4 cm in length at the sigmoid descending junction with ulceration 40 cm from the anal verge, that was bleeding on touch. A biopsy was taken, and a polypectomy for two small polyps in the ascending colon was done.

On the second day of admission, an echocardiogram was done which revealed a thickened anterior mitral valve leaflet with a mass attached to the anterior mitral valve leaflet. The mass was 4x7 mm in size and moved to and from across the mitral valve. The ECG also showed ruptured chorda vegetation on the anterior mitral valve leaflet, moderated eccentric mitral regurgitation, ejection fraction (EF) of 40%-45%, and mild left ventricular systolic dysfunction. The echocardiogram report suggested infectious endocarditis, hence the cardiology team requested blood culture. Histopathology for the colonic mass returned as moderately differentiated adenocarcinoma. The polyps were inflamed, tubular, and adenomatous with mild dysplasia.

**Figure 1:**
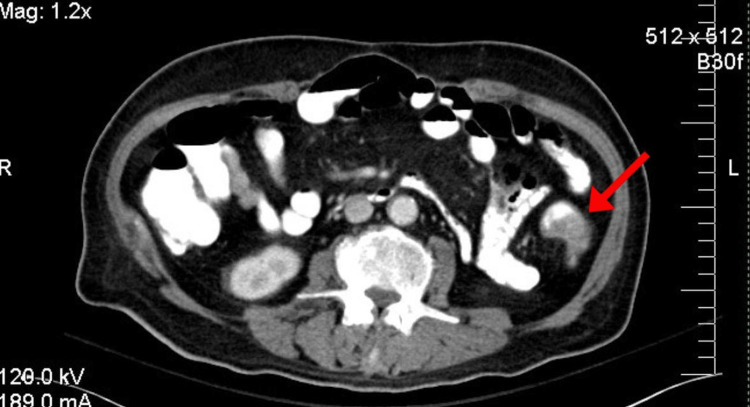
Axial view CT Abdomen with IV contrast. Mural circumferential thickening at the at inferior portion of the descending colon near the distal sigmoid 3.5 cm in length.

**Figure 2:**
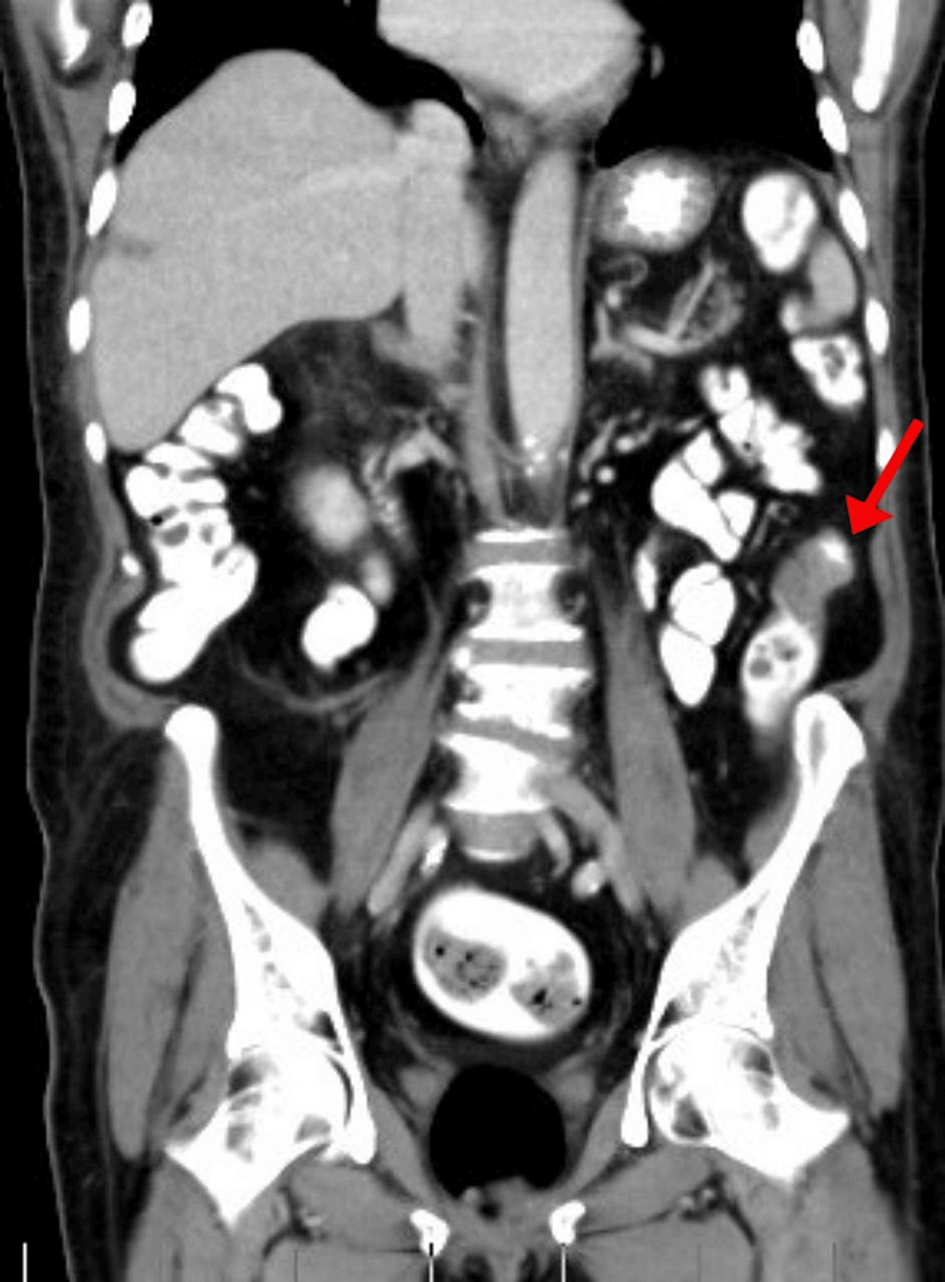
Coronal view CT Abdomen with IV contrast. Mural circumferential thickening at the at inferior portion of the descending colon near the distal sigmoid 3.5 cm in length.

A CT scan of the chest, abdomen, and pelvis with (CT CAP) with IV contrast was performed for staging purposes. This showed evidence of a short segment of mural circumferential thickening at the inferior portion of the descending colon near the distal sigmoid 3.5 cm in length no pericolonic soft tissue invasion and sign of metastasis disease (Figures 1, 2).

The patient continued to have blood loss and received another four units of PRBCS. After three days the patient became hypotensive and was shifted to the coronary care unit (CCU) with a diagnosis of non-ST-elevation myocardial infarction (NSTEMI) complicated by cardiogenic shock. Cardiac catheterization was performed. Unfortunately, the patient still had continuous bleeding and received another four units of PRBCS and a decision to conduct left hemicolectomy as an emergency surgery was taken. The patient stayed in the hospital to continue antibiotics (ceftriaxone 1 gram, intravenous, every 12 hours for six weeks), and also after that cardiac catheterization was done and a stent inserted.

Final histopathology came as moderated differentiated adenocarcinoma 2.5 cm, all margins free of tumor, P T3 N0 M0. The patient was discharged in good condition and followed up for two years with the colorectal team clinic. At the last visit six months back, the patient was doing well.

## Discussion

Colorectal cancer can be prevented with the help of screening, which happens by early detection and removal of adenomatous polyps (which can be precancerous). However, if CRC is detected early, it shows a significant survival rate [1]. A study conducted by Corredoira et al., about the effects of organized CRC screening on cancer incidence and mortality, showed a decrease in the following: the incidence of CRC by 25.5%, the development of advanced-stage CRC by 36.2%, and the mortality rate by 52.4% [2]. A CRC screening option review published by Baddour et al. states that colonoscopy is the most appropriate test for those who have a high risk of developing CRC [3]. 

Many case studies reported a relationship between gastrointestinal organisms (E. faecalis & S. bovis) and developing infective endocarditis. The translocation of these organisms toward the bloodstream is linked to the presentation of damage in colonic mucosa (increasing the permeability) that might result from infection, ischemic and inflammatory colitis, or secondary bleeding as a consequence of tumor mucosal invasion. As there is a well-established relationship between S.bovis infective endocarditis and CRC, It is highly recommended to do a colonoscopy for them to detect CRC. On the other hand, the relationship between E. faecalis infective endocarditis and CRC is not well established yet, especially when the source of E. faecalis is unknown and can be from the genitourinary system. The biggest problem that limits colonoscopy’s role in E. faecalis infective endocarditis is that most of them are sick and cannot withstand the fluid overload with valve regurgitation needed for colonoscopy preparation. That is why it is still controversial. But if colonoscopy is feasible, it should be considered and done to detect CRC in the early stage (as many reports suggest a relation between E. faecalis infective endocarditis and CRC) and treat it as early as possible [4-6]. 

A case was published in 2022 of a 69-year-old woman with diabetes mellitus (DM), hypertension (HTN), hypersensitivity pneumonitis, obesity with gastric band placement, and right breast cancer status post-lumpectomy. She presented to the ER complaining of shortness of breath, chest pain, and diaphoresis. ECG showed anterior ST elevation and high troponin that confirmed myocardial infarction. The patient was shifted immediately after resuscitation to the cardiac catheterization laboratory for intervention. Post-procedure, the patient had a shock and was investigated. The patient’s transesophageal echocardiogram showed a significant vegetation mitral valve, confirming the presence of infective endocarditis, and blood cultures confirmed E. faecalis bacteremia. Results of the investigation that looked for the source of bacteremia, colonoscopy showed a perforation 20 cm from the anal verge at the rectosigmoid junction. The cause of myocardial infarction is suggested to be a septic embolus originating from her mitral valve vegetation [7]. 

A research conducted on 25 patients with S. bovis-group infective endocarditis underwent a gastroenteroscopic evaluation using a gastroscopy, colonoscopy, and CT colonography to check for gastrointestinal neoplasia following surgery. About 84.4 % of patients who obtained this assessment in the study had colorectal neoplasia [1]. 

In our case, after starting to manage the infection, the patient became hypotensive and was diagnosed with NSTEMI, which was complicated by cardiogenic shock. NSTEMI and AKI might result from a septic embolic, as suggested in the previous case.

## Conclusions

Since there is a strong association between S. bovis infective endocarditis and CRC, further studies are needed to confirm the relationship between E. faecalis infective endocarditis and CRC, and potentially a screening program for those patients, if approved.
